# Deciphering Human Cell-Autonomous Anti-HSV-1 Immunity in the Central Nervous System

**DOI:** 10.3389/fimmu.2015.00208

**Published:** 2015-05-08

**Authors:** Fabien G. Lafaille, Michael J. Ciancanelli, Lorenz Studer, Gregory Smith, Luigi Notarangelo, Jean-Laurent Casanova, Shen-Ying Zhang

**Affiliations:** ^1^Rockefeller Branch, St. Giles Laboratory of Human Genetics of Infectious Diseases, The Rockefeller University, New York, NY, USA; ^2^The Center for Stem Cell Biology, Developmental Biology Program, Sloan-Kettering Institute for Cancer Research, New York, NY, USA; ^3^Department of Microbiology-Immunology, Northwestern University Feinberg School of Medicine, Chicago, IL, USA; ^4^Division of Immunology, Boston Children’s Hospital and Harvard Medical School, Boston, MA, USA; ^5^Howard Hughes Medical Institute, New York, NY, USA; ^6^Laboratory of Human Genetics of Infectious Diseases, Necker Branch, INSERM U1163, Necker Hospital for Sick Children, Paris, France; ^7^Imagine Institute, Paris Descartes University, Paris, France; ^8^Pediatric Hematology-Immunology Unit, Necker Hospital for Sick Children, Paris, France

**Keywords:** HSV-1, CNS, disease modeling, antiviral immunity, hiPSCs, HSE, interferons, TLR3

## Abstract

Herpes simplex virus 1 (HSV-1) is a common virus that can rarely invade the human central nervous system (CNS), causing devastating encephalitis. The permissiveness to HSV-1 of the various relevant cell types of the CNS, neurons, astrocytes, oligodendrocytes, and microglia cells, as well as their response to viral infection, has been extensively studied in humans and other animals. Nevertheless, human CNS cell-based models of anti-HSV-1 immunity are of particular importance, as responses to any given neurotropic virus may differ between humans and other animals. Human CNS neuron cell lines as well as primary human CNS neurons, astrocytes, and microglia cells cultured/isolated from embryos or cadavers, have enabled the study of cell-autonomous anti-HSV-1 immunity *in vitro*. However, the paucity of biological samples and their lack of purity have hindered progress in the field, which furthermore suffers from the absence of testable primary human oligodendrocytes. Recently, the authors have established a human induced pluripotent stem cells (hiPSCs)-based model of anti-HSV-1 immunity in neurons, oligodendrocyte precursor cells, astrocytes, and neural stem cells, which has widened the scope of possible *in vitro* studies while permitting in-depth explorations. This mini-review summarizes the available data on human primary and iPSC-derived CNS cells for anti-HSV-1 immunity. The hiPSC-mediated study of anti-viral immunity in both healthy individuals and patients with viral encephalitis will be a powerful tool in dissecting the disease pathogenesis of CNS infections with HSV-1 and other neurotropic viruses.

## Introduction

Herpes simplex virus 1 (HSV-1) is a double-stranded DNA virus of the α-herpesvirinae subfamily (1). These viruses are characterized by a short replication cycle and their ability to establish latency in sensory neurons (2). The proportion of individuals infected with HSV-1 worldwide increases with age and can reach up to 90% in the elderly (3, 4). Primary infections by this virus lead to a life-long latency (5) although reactivation of the virus can occur in infected individuals, because of stress or exposure to ultraviolet radiation, leading to anterograde spread of HSV-1 to epithelial cells and formation of herpes labialis. Encephalitis is a rare manifestation of HSV-1 infection and has been reported in an estimated 1 per 250,000 cases per year (1). Approximately one-third of cases are caused by primary infection in children (6). Herpes simplex encephalitis (HSE) is the most common sporadic viral encephalitis in the western countries, accounting for 20% of the 20,000 annual cases in the Unites States (7–9). Most patients show positive PCR results for HSV-1 DNA in their cerebral spinal fluid. Although >70% of patients survive the disease with acyclovir treatment, an inhibitor of viral DNA synthesis (10), the majority of them consequently suffer from epilepsy, mental retardation, and other neurological deficits (11).

Herpes simplex virus 1 reaches the central nervous system (CNS) via cranial nerves such as olfactory and trigeminal neurons (1). During the course of HSE, no viremia is detected in affected patients. Within the brain, destruction of glial and neuronal cells is detectable during the first month of disease (12), suggesting a cytotoxic effect of HSV-1 on both neurons and astroglial cell types. The fact that most HSE patients have intact T- and B-cell responses to HSV-1 and are otherwise healthy (13) suggests that intrinsic immunity to HSV-1 by CNS cell types may be a key in controlling viral spread within the CNS. Viral susceptibility and production of protective cytokines, mainly interferons (IFNs), in the CNS of infected hosts during the course of HSV-1 infection have been studied *in vivo* using mouse models, and reviewed elsewhere (13–15). Interestingly, induction of IFNs and other cytokines because of viral infection differs between human and mice (16). Hence, human CNS cell-based models of anti-HSV-1 immunity have become increasingly relevant. The disease pathogenesis of HSE has long remained unclear, until the recent findings that inborn error of toll-like receptor 3 (TLR3) immunity may underlie the development of HSE in children with mutations in *TLR3*, *UNC93B1*, *TRIF*, *TRAF3*, and *TBK1* (14). The authors herein review the studies of human CNS anti-HSV-1 immunity based on *in vitro* models of human cell lines and primary cells, as well as their recent study using human induced pluripotent stem cells (hiPSCs)-derived neuronal cells from patients with HSE-causing TLR3 pathway deficiencies.

## Anti-HSV-1 Immunity in Neuron Cell Lines and Primary Neurons

The human embryonic carcinoma cell line NT2 has been used as an *in vitro* model in studies of CNS neurons anti-HSV-1 immunity. Characterized as an equivalent of CNS neuronal progenitors, NT2 cells are capable of differentiating into neuron-like cells in response to retinoic acid (RA) treatment (17, 18). Differentiation of these progenitor cells is very efficient, giving rise to 99% pure populations of “NT2-N” neurons, which morphologically resemble human primary neurons (19). These cells can be infected by HSV-1 which induces mRNA for IFN-α and IFN-λ1 (20). NT2-N cells also express TLR3 mRNA, which is a receptor capable of recognizing double-stranded RNA (dsRNA) generated during HSV-1 infections (21–23). The synthetic dsRNA polyinosinic-polycytidylic acid [poly(I:C)], used as an agonist for TLR3 (24), induces IFN-α and IFN-β (type I IFNs) as well as IFN-λ1 (type III IFN) expression in NT2-N neurons in a dose-dependent manner (19–21). Similar poly(I:C) responses were observed in BE(2)-C/m cells differentiated on RA treatment to mature neurons (25). Pre-treatment with poly(I:C) reduces HSV-1 replication by 80% in NT2-N neurons, down to similar levels as observed in IFN-α, IFN-β, IFN-λ1, and -λ2 pre-treated cells. Interestingly, HSV-1 infection in NT2-N cells induces IFN-α but not -β expression, although the neurons can respond to this cytokine (21). Overall, these data suggested that human CNS neuronal-like cells harbor a functional TLR3/IFN system involved in anti-HSV-1 immunity.

To investigate the role of the TLR/IFNs pathway against HSV-1 in a physiologically more relevant model, human primary CNS neurons have been cultured/isolated from aborted fetuses’ cortical tissues (19, 20, 26). Neuronal cultures ultimately consist of 80–90% neurons and 10–20% astroglial cell types. TLR2 and TLR3 are expressed throughout the CNS (27), including in human primary neurons (28). Like in NT2-N cells, poly(I:C) can induce elevated levels of IFN-α, IFN-β, and IFN-λ1 in primary neurons. Pre-treatment with IFN-λ1 or -λ2 reduces HSV-1 replication in these cells and induces IRF7 expression, a key transcription factor creating a positive feedback loop for IFN production (29). Globally, these results indicated that the TLR3/IFN pathway is also critical for control of HSV-1 virus infection in primary CNS neurons. It would also be important to look at anti-HSV-1 immunity in human peripheral nervous system (PNS) neurons, as HSV-1 spreads along nerves of the PNS on its way to the CNS leading to HSE, as shown by the presence of late viral proteins in PNS neurons (30). There is, however, no reported cell line capable of generating PNS neurons such as trigeminal neurons. Hence, dorsal root ganglia explants from human fetal tissues have been utilized as *in vitro* models (31–33). Although HSV-1 can infect PNS neurons, their response to infection was not studied. In addition, trigeminal ganglia (TG) removed from cadavers and latently infected by HSV-1 showed elevated transcription of IFN-γ (34). Further studies will need to address the response of purified human TG neurons to primary infection with HSV-1.

## Anti-HSV-1 Immunity in Primary Glial Cells

Central nervous system glial cells include astrocytes, oligodendrocytes, and microglial cells. Within the CNS, astrocytes are the most abundant cell type (35). Culture of fetal tissues in medium containing fetal bovine serum generates >90% pure primary human astrocytes population upon passaging (36, 37). These cells express primarily TLR2 and TLR3, the latter mainly on their plasma membrane (27). *In vitro* poly(I:C) stimulation induces human astrocytes to express IFN-λ1, -λ2/3 (38), and IFN-β, but not TNF-α (36, 39), a cytokine controlling HSV-1 reactivation rate in mice (40). Numerous other IFN stimulated genes can be upregulated through poly(I:C) stimulation of primary human astrocytes, including IL-6, IRF7, IFIT2, and MX1. These cells are also permissible to HSV-1 infection (41). Recombinant IFN-λ1 and -λ2 can block HSV-1 replication in human astrocytes and induce the expression of endogenous IFN-α through which they exert their anti-viral activity (26). These data suggest that IFN-λ and IFN- α have a role in anti-HSV-1 immunity at least in astrocytes.

Although human primary oligodendrocytes are known to primarily express TLR2 and TLR3 similar to astrocytes (27), no human primary oligodendrocyte culture system exists to date for the study of antiviral immunity. Microglia cells clear the CNS of dead neurons via phagocytosis (42). In contrast to neurons and astrocytes, primary human microglia cells express a wide range of TLRs, but presence of TLR3 proteins was only observed in intracellular vesicles as assessed by immunostaining (27). Furthermore, these cells are not permissive to HSV-1 replication, although immediate early viral proteins are present in infected cells (41). Microglia cells also undergo apoptosis quickly following infection. Interestingly, although primary human microglial cells stimulated by HSV-1 do not upregulate IFN-α and -β expression levels (41), they do display elevated levels of NF-κB activation and TNF-α production (43). TNF-α pre-treatment blocks HSV-1 replication in primary human astrocytes but not in primary human neurons suggesting a cell-type specific impact of this microglia-secreted anti-viral cytokine. The potential role of TNF-α in anti-HSV-1 immunity is highlighted by the fact that treatment with inhibitors of TNF-α was associated with HSE susceptibility in several individuals (44).

## Anti-HSV-1 Immunity in hiPSC-Derived Neuronal Cells

In order to study the HSE pathogenesis in patients with known disease-causing genetic etiologies, we have recently established an hiPSC-based *in vitro* model of HSV-1 infection in CNS-resident cells (45). Advances in the field of stem cell biology has allowed for the reprogramming of human somatic cells to a pluripotent state (46). Human pluripotent stem cells have two key properties: they can differentiate into any cells of the three germ layers (ectoderm, endoderm, mesoderm) and self-renew, potentially replicating indefinitely in culture. Hence, we have reprogrammed dermal fibroblasts from HSE patients to iPSCs and tested anti-HSV-1 immunity in CNS-resident cells derived from these iPSCs. After differentiating healthy control and patient-specific iPSCs toward highly enriched populations of forebrain CNS neurons, astrocytes, oligodendrocyte progenitor cells (OPCs), and neural stem cells (NSCs), we have assessed viral susceptibility and IFNs production in each cell type. Poly(I:C) treatment induced elevated levels of IFN-λ1 mRNA in OPCs, and IFN-β as well as -λ1 in neurons and astrocytes from healthy donors. Pre-treatment of poly(I:C) as well as of recombinant IFN-α and -β inhibited HSV-1 replication in these cell types.

We then focused on cells from two genetically defined HSE patients, harboring an autosomal recessive complete TLR3 (47) or UNC-93B (48) deficiency. UNC-93B is a key factor within the TLR3/IFN pathway required to activate downstream molecular signals (49). Susceptibility to HSV-1 was shown to be cell type and genotype dependent. Indeed, healthy neurons and OPCs could control viral replication, whereas UNC-93B- or TLR3-deficient cells could not. This cellular phenotype was related to the impaired production of IFN-β or -λ1 in response to HSV-1 or poly(I:C) in the same cells. On the other hand, no differences were observed between control and patients’ NSCs and astrocytes. Hence, these *in vitro* experiments demonstrated that CNS neurons and OPCs control HSV-1 infection intrinsically in a TLR3-dependent manner, although the *in vivo* protective role of astrocytes and NSCs cannot be excluded. Overall, these iPSC-based experiments are in agreement with studies based on cell lines and primary sources, suggesting that TLR3 and IFN responses play important roles in defense against HSV-1 infection in CNS cells (Table 1). HSE is, to our knowledge, the first example of hiPSC-mediated modeling of an inborn error of immunity, demonstrating a disease-relevant immunological cellular phenotype in disease-relevant CNS cell types.

**Table 1:** **Selected cytokines properties in human CNS cell types**.

Cytokine	Stimulation	Cell line-derived	Primary cells			Control hiPSC-derived		
		NT2 neurons	Neurons	Astrocytes	Microglia	Neurons	Astrocytes	OPCs
	Poly(l:C)	↑	↑	×	×	n/a	n/a	n/a
IFN-α	HSV-1	↑	↑	n/a	n/a	n/a	n/a	n/a
	Viral protection	✓	✓	✓	n/a	✓	✓	✓
	Poly(l:C)	↑	↑	↑	×	↑	↑	n/a
IFN-β	HSV-1	×	↑	n/a	n/a	↑	↑	n/a
	Viral protection	✓	✓	✓	n/a	✓	✓	✓
	Poly(l:C)	↑	↑	↑	n/a	n/a	↑	↑
IFN-λ1	HSV-1	n/a	n/a	n/a	n/a	n/a	↑	↑
	Viral protection	✓	✓	✓	n/a	×	×	×
	Poly(l:C)	n/a	n/a	↑	n/a	n/a	n/a	n/a
IFN-λ2/3	HSV-1	n/a	n/a	n/a	n/a	n/a	n/a	n/a
	Viral protection	✓	✓	✓	n/a	n/a	n/a	n/a
	Poly(l:C)	n/a	n/a	×	n/a	n/a	n/a	n/a
TNF-α	HSV-1	n/a	n/a	n/a	↑	n/a	n/a	n/a
	Viral protection	×	×	✓	n/a	n/a	n/a	n/a

*↑ stimulation upregulates the cytokine*.

*× stimulation does not upregulate the cytokine or showed no protective effect against HSV-1 infection*.

*Other cytokines can be expressed in these cell types but the authors chose to summarize here those tested*.

## Conclusion

Human neuronal cell lines as well as primary cells have allowed for the study of anti-HSV-1 immunity of the human CNS. Human cell line-derived and primary CNS neurons express TLR3, display elevated levels of IFN-α, -β, and -λ1 on poly(I:C) treatment, and are permissible to HSV-1 infection. Interestingly, although poly(I:C), IFN-α and -β can block viral replication in primary, cell line-derived and control iPSC-derived human neurons, IFN-λ1 and -λ2 could only do so on primary and cell line-derived neurons. Like oligodendrocytes, human primary astrocytes express TLR3 and were found to express high levels of IFN-λ1, -λ2/3, as well as IFN-β on poly(I:C) stimulation. Primary astrocytes are permissible to HSV-1 infection and are protected from viral replication by IFN-λ1 and -λ2, which was not the case for control iPSC-derived astrocytes. Interestingly, we were able to compare directly the susceptibility to HSV-1 of control hiPSC-derived CNS neurons, OPCs, and astrocytes and demonstrated that neurons and OPCs have an intrinsic resistance to viral replication. This control of HSV-1 replication was lost in neurons and OPCs derived from patient-specific iPSCs with inborn errors of TLR3 pathway immunity. Overall, these data suggest a key role for the TLR3–IFN pathway in cell-autonomous HSV-1 immunity in neurons and OPCs, and a potential role for this pathway in astrocytes, although this may not be the case in microglia cells. Indeed, these cells do not express TLR3 on their surface, are not permissible to HSV-1 replication, and do not upregulate IFN-α and -β on HSV-1 infection, although they do express the cytokine TNF-α, which can block HSV-1 replication in human astrocytes but not in neurons.

Obviously useful, experiments in primary human cells are however limited because of the difficulty to access cell sources, embryos, and cadavers. Furthermore, some cells are either difficult to purify, such as TG neurons, or simply not possible to isolate, such as oligodendrocytes. The use of hiPSCs can alleviate these issues by providing an unlimited source of neuronal cell types for disease modeling (50). Within the last 6 years, at least 190 studies have described iPSC-based human disease models (51). Disease modeling using hiPSCs has benefited from key technical developments including the establishment of differentiation protocols mimicking early embryonic development steps to generate CNS (52, 53) and PNS (54, 55) cells. Furthermore, the advent of non-integrating methods of reprogramming (56) and new technological breakthroughs in the field of gene editing (57, 58) allows for the precise relationship assessment between germline mutations and disease phenotypes. However, some tools are still missing to completely survey the response of all neuronal cells types infected by HSV-1 during the course of HSE. Indeed, to date, no protocol exists for the generation of microglia cells from hiPSCs. Hence, while a work in progress, *in vitro* hiPSC-based model of HSV-1 infection in CNS cells holds tremendous potential for our understanding of the molecular and cellular basis of cell-autonomous, non-hematopoietic, anti-HSV-1, intrinsic immunity in the CNS.

## Conflict of Interest Statement

The authors declare that the research was conducted in the absence of any commercial or financial relationships that could be construed as a potential conflict of interest.
